# Trogocytosis and Allergy

**DOI:** 10.3390/cells15060516

**Published:** 2026-03-13

**Authors:** Olga Sergeevna Boeva, Veronika Sergeevna Abbasova, Vladimir Aleksandrovich Kozlov, Ekaterina Aleksandrovna Pashkina

**Affiliations:** 1 Research Institute of Fundamental and Clinical Immunology, 14, Yadrintsevskaya St., 630099 Novosibirsk, Russia; 2 Department of Clinical Immunology, Novosibirsk State Medical University, 52, Krasny Prospect, 630091 Novosibirsk, Russia

**Keywords:** trogocytosis, allergy, type2-mediated immunity, antigen presentation

## Abstract

**Highlights:**

**What are the main findings?**

- Trogocytosis is a cell interaction process that influences immune responses in health and disease, including allergies.
- Trogocytosis affects immune balance.

**What are the implications of main findings?**

- Trogocytosis may shift immune responses toward T2 and contribute to the development of T2 allergic diseases.
- Trogocytosis may also contribute to the development of tolerance to an allergen.

**Abstract:**

Trogocytosis is the process of engulfment of a portion of a cell’s membrane by another cell. This process is characterized by the transfer of membrane fragments and proteins between adjacent cells without their complete fusion or phagocytosis, which distinguishes it from classical cellular uptake pathways. In the immune system, the initiating signal for trogocytosis is antigen presentation or the interaction of the Fc receptor with an antibody bound to the cell. During trogocytosis, T cells transfer not only the MHC molecule with the antigenic peptide, but also the costimulatory molecules CD80, CD86, OX-40 and others. As a result of trogocytosis, cells can transfer various surface molecules, acquire new immunological properties, and modulate each other’s activity. This review examines the basic mechanisms of trogocytosis, the involvement of T2-mediated immunity components in trogocytosis, and its possible role in allergies.

## 1. Introduction

In the mid-20th century, scientists noticed a process they described as the “stealing” of surface molecules [1]. This phenomenon was first described in amoebae, and later between cells of the immune system [2,3,4,5]. The observed process became known as partial phagocytosis, cannibalistic gnawing, extraction, biting off, acquisition, since in translation from the Greek (trogo-) means “nibble” or “to bite off”, and the transfer of membrane proteins was observed between cells [2]. The concept of “trogocytosis” was first introduced into scientific circulation by T. Brown. He discovered that Naegleria fowleri, known as the “brain-eating amoeba”, engulfs mouse embryo cells piece by piece [3]. The main difference between amoebas and immune cells is that in the latter case, both cells usually remain alive [3].

Currently, a growing body of research demonstrates the important role of trogocytosis in the immune system. Trogocytosis is a process where one cell actively takes a fragment of another cell’s membrane, transferring surface molecules in the process. It is generally distinguished from phagocytosis, which engulfs the entire cell [1]. Trogocytosis is involved in antigen presentation, T-lymphocyte differentiation, nervous system remodeling, and embryonic development, and plays a role in anti-infective and anti-tumor immunity [2]. Trogocytosis transports not only major histocompatibility complex (MHC) class II molecules, but also costimulatory molecules, tumor antigens, and various pathogens. According to the literature, the following cells can participate in this process: T cells (γδ T cells and CD4^+^ and CD8^+^ αβ T cells), B cells, natural killer cells (NK), dendritic cells ((DCs), monocytes/macrophages, neutrophils, endothelial cells, fibroblasts, eosinophils, basophils, tumor cells and various pathogens. However, it is still not entirely clear what role trogocytosis plays both under physiological conditions and in pathology. Currently, researchers are focused on studying the trogocytosis of immune cells in the development of infectious diseases, oncopathology, and, partially, in autoimmune processes. Most of the works by different authors are devoted to the role of trogocytosis of CD8^+^ T-lymphocytes in the progression of tumor growth [6], the evasion of the immune response by tumor cells through trogocytosis [6], a decrease in the effectiveness of targeted antitumor therapy and specific CAR-T cells as a result of trogocytosis of tumor antigens and the MHC-T-cell receptor (TCR) complex, respectively [7,8,9]. However, the role of trogocytosis in allergic diseases is rather poorly covered in the literature.

## 2. The Main Mechanisms of Trogocytosis by Immune Cells

Trogocytosis in immune cells has been shown to be mediated by two main pathways: the formation of an immunological synapse between MHC and the TCR (MHC-TCR) and the interaction of the immunoglobulin receptor with its ligand (FcR-Ig) [10,11,12]. The first pathway has a far greater impact on the immune response, as it can involve CD4^+^ and CD8^+^ T lymphocytes, antigen-presenting cells (APCs), NK, and basophils. The second pathway can be used by B lymphocytes, NK cells, macrophages and neutrophils. According to the literature, several mechanisms of trogocytosis are distinguished: trogocytosis caused by the interaction of TCR–MHC signaling mechanism, cross-dressing as a mechanism of trogocytosis, the mechanism of trogocytosis in neutrophils, leading cells to apoptosis.

### 2.1. TCR–MHC-Mediated Trogocytosis

Trogocytosis involves interaction between a ligand and a receptor, such as the TCR of T lymphocytes with the pMHC (peptide-associated major histocompatibility complex) complex located on the surface of APCs. Upon contact between APCs and T cells, an immunological synapse is formed, which then activates T cells and the pMHC, along with fragments of APC membranes, are translocated to the surface of T cells [1]. Studies show that by acquiring peptide–MHC class II (pMHC-II) complexes from the surface of APCs through trogocytosis, CD4^+^ T cells support TCR signaling and promote CD4^+^ T cell survival even after APC removal. This mechanism leads to full activation and stimulation of T lymphocytes by antigen. In contrast to other processes, such as phagocytosis, trogocytosis prolongs the interaction time between the T cell and the pMHC [3]. CD4^+^ T cells take up pMHC-II from APCs, while CD8^+^ T cells nibble off peptide–MHC class I (pMHC-I) complexes from APCs. CD4^+^ T cells can also acquire both cognate pMHC-II and bystander pMHC-I, possibly due to the localization of the bystander pMHC-I near cognate pMHC-II. Similarly, CD8^+^ T cells can acquire both cognate pMHC-I and bystander pMHC-II [1]. Other molecules have been shown to be transferred along with pMHC, including costimulatory molecules (CD80, CD86, and OX-40) and integrin ligands (e.g., ICAM-1) [13]. Thus, after acquiring the MHC-TCR complex and associated costimulatory molecules, the trogocytized cell (trogocyte) acquires new properties.

It has been shown that CD4^+^CD80^+^CD86^+^ cells are capable of performing an antigen-presenting role in relation to other, non-activated CD4^+^ T-lymphocytes [14,15,16]. Zhou et al. [17] demonstrated that the trogocytosis of pMHC and CD80 from APCs to T cells sustains T cell activation and proliferation even in the absence of APCs. Consequently, the number of activated CD4^+^ T cells can significantly exceed the number of APCs. This can ultimately lead to interactions between new T cells and trogocytized T cells (TT interactions), but this leads to increased apoptosis and induction of anergy [17,18].

**Figure 1:**
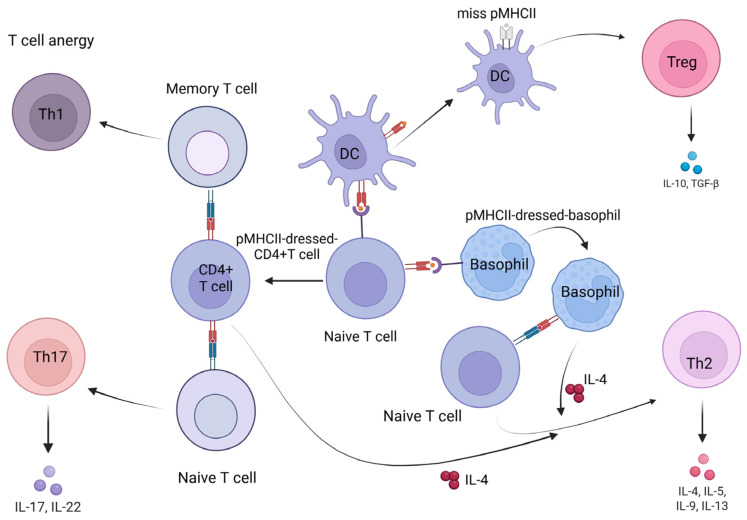
Trogocytosis and Th polarization Created in BioRender. Pashkina, E. (2026). https://BioRender.com/tkvzwq2 (accessed on 20 January 2026).

It has been shown that CD4^+^ T cells can trogocytose as T-regulatory cells and a subset of T helper type 2 (Th2) cells. Trogocytosed CD4^+^ T cells react with naive and memory CD4^+^ T cells, leading to anergy of naive CD4^+^ T cells and T helper type 17 (Th17) activation of memory CD4^+^ T cells [1,19]. Regulatory T cells (Tregs) are capable of depleting CD80/CD86 molecules on APCs by nibbling them through CTLA-4-dependent trogocytosis [20]. It has been shown that CTLA-4-positive cells can nip CD80/CD86 from opposing cells, destroying them and leading to impaired costimulation via the CD28 molecule [21]. Depletion or blockade of CD80 through CTLA-4-dependent trogocytosis leads to an increase in free programmed death-ligand 1 (PD-L1). Thus, Tregs may exert dual suppressive effects by limiting CD80/CD86 and upregulating free PD-L1 on APCs [22]. To enforce immune tolerance, Tregs actively use trogocytosis—more so than naïve or effector T cells—to remove MHCII and costimulatory molecules from the APC surface [20]. However, both in vitro and in vivo studies have shown that trogocytosis-mediated signaling influences CD4^+^ T cell differentiation and effector cytokine production and may play a role in enhancing or shaping a T2 immune response [22]. The role of trogocytosis in the polarization of the immune response is shown in Figure 1.

Unlike CD4^+^ and CD8^+^ αβT cells, γδT cells can bind not only to pMHC but also to other ligands via the TCR [23]. γδT cells have been shown to trogocytose tumor cells [24]. The main subset of gamma delta T cells, Vγ9Vδ2 cells, are MHC-independent and recognize phosphorylated antigens presented by butyrophilin 3A1/2A1 (BTN3A1/BTN2A1) [25]. Schneiders et al. showed that Vγ9Vδ2 T cells, by acquiring the CD1d molecule through TCR-mediated trogocytosis, can activate iNKT cells [26].

Thus, TCR-pMHC-mediated trogocytosis, on the one hand, can lead to an increase in APCs, and as a consequence, an increase in antigen presentation and an enhancement of the immune response, but, on the other hand, can lead to a weakening of T-lymphocyte activation and the loss of MHC and costimulatory molecules by APCs.

### 2.2. Cross-Dressing

Cross-dressing is an additional trogocytosis mechanism used by APCs to present antigen to CD8^+^ T cells [3]. This mechanism involves the translocation of pMHC-I from the surface of infected, dead, or tumor cells to APCs, particularly DCs [1,6]. As a result, “dressed DCs” (cross-dressed) activate CD8^+^ T cells without prior antigen treatment [27]. Cross-dressed DCs play a major role in the activation of CD8^+^ T cells in viral infection models and during allograft rejection [1].

The conventional DCs are further subdivided into DC type 1 cells (DC1s) and DC type 2 cells (DC2s) [28,29]. DC2s process extracellular antigens for presentation solely on MHC-II. In contrast, cDC1s can direct the same antigens into both the conventional MHC-II pathway and the cross-presentation pathway for MHC-I [30]. According to the literature, cross-dressing has been shown to occur by both DC1 and DC2, but DC2s exhibit a higher ability to cross-dress neighboring DC-derived MHC-I. This difference may be related to the donor cells from which DCs acquire MHC-I.

MHC can also be transferred between DCs via cross-dressing [30,31,32]. DCs are professional APCs that play a key role in the primary immune response. These cells can exchange antigen within the MHC-I and then present the peptide to T cells. Using this transfer mechanism, DCs accelerate the immune response, as they do not encounter the antigen and do not process it, thus accelerating T cell activation presenting cells.

### 2.3. FcγR–Mediated Trogocytosis

Fcγ receptor (FcγR)-mediated trogocytosis occurs through the recognition of IgG immune complexes on donor cells by FcγR on acceptor cells [10]. This engagement leads to the formation of an immunological synapse between the acceptor and IgG-opsonized donor cells, enabling the trogocytic transfer of membrane patches containing IgG and antigen to the effector cell. This recognition is mediated by FcγR, which is expressed on various immune cells, including macrophages, neutrophils, NK cells, and mast cells (MCs) [33].

#### 2.3.1. Neutrophils

Neutrophils are the most numerous type of leukocyte in circulating blood [34]. Neutrophils are capable of not only phagocytosis but also trogocytosis [35]. Unlike other cells, neutrophils act as a group to “nibble off” pathogen particles and induce apoptosis. The excessive removal of membrane via trogocytosis may trigger cell death, a phenomenon specifically termed trogoptosis (trogocytosis-mediated apoptosis).

Neutrophils most often use trogocytosis to combat protozoa, such as Trichomonas vaginalis [5]. In addition, neutrophils can eliminate opsonized tumor cells through a process known as trogoptosis [36]. FcγR, in combination with CD11b/CD18 integrins, enhances tumor cell activity by blocking CD47-SIRPα interactions. CD47-SIRPα is a transmembrane protein found on red blood cells that prevents their uptake by macrophages. Neutrophils are thus able to enhance ADCC (antibody-dependent cell-mediated cytotoxicity) of tumor cells [37]. Neutrophils are one of the most numerous populations of innate cells involved in the pathogenesis of rheumatoid arthritis (RA). This subset is located primarily in synovial fluid and, to a lesser extent, within the synovial tissue. Functionally, neutrophils from patients with RA differ from those of healthy donors by possessing the greatest cytotoxic potential and undergoing rapid activation in the presence of reactive oxygen species (ROS). Granules contained in the cytoplasm can be released in response to antibodies to anticitrullinated protein (ACPA) and rheumatoid factor (RF) in the joint of patients with RA via the FcγR receptor [38]. Trogocytosis has also been detected in the neutrophil population, with CD8 and additional TCR and CD3 transferred from T cells as a result of FcγR-mediated trogocytosis by neutrophils. In line with this model, Masuda et al. studied this process in autoimmune diseases. Neutrophils, by binding antibodies, can trogocytose various molecules, particularly CD8, TCR, and CD3. Using this principle, the study demonstrated that neutrophils can remove excess autoantibodies, which may be a protective mechanism in autoimmune diseases such as RA. (Human anti-mouse IgG antibodies in serum increase FcγR-mediated trogocytosis.) Thus, it was concluded that lower endocytic activity by neutrophils may contribute to the development of autoimmune diseases, as antibody utilization is impaired [39].

#### 2.3.2. Macrophages

Also, antibody therapy enhance tumor cell death through macrophage trogocytosis [40]. However, macrophage trogocytosis does not always result in cell death; sometimes, this process is used for regulation. Trogocytosis by macrophages serves distinct functions in different tissues: in the bone marrow, it tags hematopoietic stem cells for retention [41], while in the developing brain, microglia employ it to prune synaptic connections [42].

Following administration of monoclonal antibodies (MAbs), FcγR-expressing cells, including monocytes, macrophages, neutrophils, and NK cells, can cleave MAb-associated cell surface molecules from target cells (tumors) via trogocytosis. This ultimately leads to decreased efficacy of MAb-based therapy [37]. Rituximab infusion in CLL patients reduces CD20 expression on tumor cells via trogocytosis, leading to incomplete tumor cell lysis and subsequent resistance to therapy. A study by Williams et al. suggested low-dose anti-CD20 therapy to reduce the effect of trogocytosis and enhance clearance of circulating CLL cells [43].

#### 2.3.3. NK Cells

NK cells also possess the capacity for trogocytosis; their key marker is CD16, a low-affinity receptor for immunoglobulin G (FcγRIII) [44]. Consequently, NK cells can also participate in the destruction of cells surrounded by antibodies. However, NK cell trogocytosis does not lead to an enhanced antitumor response [45,46]. For example, when NKG2D and MICA bind, trogocytosis is triggered, resulting in NK cells acquiring MICA from donor or tumor cells. NK cells that have cleaved MICA can interact with NKG2D on NK cells and trigger NK cell fratricide. A similar fratricidal mechanism occurs when NK cells trogocytose the Rae-1 protein, another ligand for NKG2D [47]. Similar to T cells, NK cells can acquire the non-classical MHC molecule HLA-G via trogocytosis. HLA-G1 on NK cells can binds to ILT2 on neighboring NK cells, thereby suppressing their proliferation and cytotoxic function.

Additionally, NK cells can acquire CD9 (a tetraspondin family protein) through trogocytosis and suppress the cytotoxicity of these cells, as studied in high-grade tubo-ovarian serous carcinoma (HGSC). Anti-CD9 antibodies restored the cytotoxic function of NK cells. Similarly, NK cells can cleave programmed cell death 1 (PD-1) from C1498 leukemia cells through trogocytosis via the SLAM receptor and suppress their antitumor activity [47,48,49]. Like CD9, this impairment can be reversed by anti-PD-1, thereby enhancing NK cell cytotoxicity against tumors. NK cells can acquire molecules not only from tumor cells but also during interactions with other cells, such as APCs. Through trogocytosis, NK cells can acquire MHCII from DCs and, when coated with pMHC, present antigen to CD4^+^ T cells. However, this antigen presentation is inefficient and fails to activate T cells due to the absence of necessary costimulatory signals [47,48]. Furthermore, NK cells can cleave the chemokine receptor CCR7 (a receptor involved in lymph node homing) from allogeneic DCs and T cells, promoting NK cell migration to secondary lymphoid organs.

## 3. T2 (Type 2) Mediated Immunity Involves Key Cells and Trogocytosis

Type 2 immunity underlies allergic diseases such as asthma, allergic rhinitis, chronic rhinosinusitis, food and drug allergies, and atopic dermatitis [50]. Type 2 immunity, which evolved to protect against parasites and toxins, fulfills several critical functions: it expels helminths from internal tissues, maintains the integrity of microbe-rich epithelial barriers, and balances the potentially destructive effects of Type 1 inflammatory responses. Type 2 immunity consists of innate lymphoid cells type 2 (ILC2), cytotoxic T cells type 2 (TC2), and Th2 cells producing IL-4, IL-5, and IL-13, which induce MCs, basophil, and eosinophil activation, as well as B cell activation and IgE antibody production [51]. According to literature data, most cells involved in T2 immunity, with the exception of eosinophils, can participate in trogocytosis [52]. However, in the work of S. Andreone it was shown that eosinophils carry out trogocytosis of tumor cells, capturing PD-1 and TIGIT molecules [53]. Presumably, eosinophils can also trogocytose checkpoints during the immune response to an allergen, regulating the immune response.

It is known that basophils can acquire a pMHC-II from the surface of DCs during trogocytosis. Due to the ability of basophils to produce IL-4, this allows them to influence the differentiation of CD4^+^ T-naive cells toward Th2 [54]. Basophils have demonstrated the ability to induce Th2 cell differentiation in an experimental model of allergic immune response [55,56]. However, basophils are a minor subpopulation of blood granulocytes (less than 1% of blood leukocytes), which may limit the actual role of basophil trogocytosis in the development of allergic reactions.

MCs can also trogocytose, whereby MCs may receive MHC-II from DCs and can initiate T cell responses [57]. Direct contact between mast cell and DCs occurs and plays an important role in modulating the immune response [58]. Furthermore, synapses between MCs and DCs enhance antigen transfer from MCs to DCs, but not merely through trogocytosis.

In addition to producing effector cytokines, ILC2 have been shown to express MHCII in combination with the costimulatory ligands CD80, CD86, and OX40 ligand (OX40L), allowing for direct cross-talk between ILC2 and CD4^+^ T cells [59,60]. Interestingly, MHCII expression on the cell surface of ILC2s is at least partly mediated by trogocytosis [57], likely acquired from other professional APCs. It remains unclear whether costimulatory molecules are acquired through trogocytosis or transcribed by ILC2s. In co-culture experiments, ovalbumin alone, but not ovalbumin-loaded ILC2s, resulted in T cell proliferation and Th2-polarization of the immune response. It is assumed that ILC2s do not direct the T cell response by directly presenting antigen, but may do so through surface antigen exchange or trogocytosis [61]. Although ILC2s can activate T cells via antigen presentation, a reciprocal interaction exists where T cells support ILC2-driven immune responses. In vitro studies demonstrate that T cells enhance ILC2 proliferation in an antigen-dependent manner, a process correlating with T cell-derived IL-2 production [62].

## 4. The Role of Trogosytosis in Allergy

According to current concepts, an allergy is an abnormal or excessive immune response to exogenous stimuli, which can develop through various mechanisms. The original classification of hypersensitivity mechanisms underlying allergic reactions, introduced by Gell and Coombs [63], was revised in 2023 by the European Academy of Allergology and Clinical Immunology (EAACI) [64]. Nine different types were identified, including mechanisms mediated by antibodies (I–III), cells (IVa-c), barrier tissues (V), metabolites (VI), and a direct reaction to chemicals (VII), for a total of seven types, including three subtypes of cellular reactions. These types can coexist in the pathogenesis of allergic diseases.

### 4.1. Type I or Immediate Response

This type is practically identical to the anaphylactic, or reaginic, type according to the old classification. Type I allergic reactions are based on immediate reactions involving IgE, Th2, ILC2, TC2, MCs, basophils, B lymphocytes [60]. When an allergen first enters the body, the body synthesizes specific IgE, which binds to MCs or basophils. Upon repeated exposure to the allergen, the allergen binds to IgE, causing MC degranulation and the release of inflammatory mediators. Clinical examples of this type of allergic reaction include allergic rhinitis, urticaria, atopic dermatitis, and anaphylaxis [65].

The development of an allergic reaction begins with the presentation of an allergen to naive T-helpers [66]. Trogocytosis can lead to an increase in APC numbers due to DC cross-dressing and the production of pMHC and costimulatory molecules by T lymphocytes [1]. Furthermore, ILC and basophils can perform antigen-presenting functions in the T2 immunity [49,57]. On the other hand, there is reason to believe that the enhancement may not be sufficient to significantly contribute to the development of allergy. Allergen-stimulated basophils exhibit low levels of MHCII on their cell surface, significantly lower than the levels observed on B cells and DCs [67]. Furthermore, basophils did not activate antigen-specific T cell proliferation, and basophil deficiency did not reduce T cell proliferation or T2 cytokine production.

As mentioned above, CD4^+^ T cells after trogocytosis can become T-regulatory cells and Th2 cells [1,22,47]. CD4^+^ T cells, during in vitro co-culture with APCs and trogocytosis, demonstrated a decrease in IFN-γ expression from 13.4% to 1.5%, while expression of the Th2 cytokine IL-4 shifted to 77.4% [47]. Moreover, Th2-polarized CD4^+^ T cells demonstrated increased trogocytosis compared to T helper type 1 (Th1) or non-polarized CD4^+^ T cells. However, in another study, CD4^+^ T lymphocytes carrying MHC II molecules on their surface, on the contrary, are able to exert negative regulation on the Th2 immune response, suppressing the development of CD4^+^ T lymphocytes and promoting their apoptosis [17,18].

Tregs are key cells capable of suppressing the immune response to an allergen and promoting the development of tolerance in allergies. It is known that CD4^+^ T lymphocytes that perform trogocytosis are subsequently capable of differentiating into Tregs, limiting the excessive immune response [68]. Tregs themselves are also capable of trogocytosis [69,70]. Compared to naive and effector T cells, Tregs have increased trogocytotic activity [29], aimed at removing MHC class II molecules and the costimulatory molecules CD80 and CD86 from APCs, which leads to a decrease in antigen-presenting function and the induction of tolerance.

Among T cells, TCRαβ^+^CD3^+^CD4^−^CD8^−^ T cells—double-negative T cells (DNTs)—are also implicated in the pathogenesis of allergic asthma. For example, in a mouse model of OVA-induced allergic asthma, adoptive transfer of DNTs reduced lung inflammation, mucus production, and OVA-specific IgG/IgE. Murine DNTs were shown to acquire MHCII molecules from DCs via Lag3/CD223 (a CD4 homolog that binds to MHCII). DNTs may suppress the antigen-presenting activity of DCs, similar to Tregs, by capturing MHCII from the DC surface and impairing antigen presentation [71].

### 4.2. Type II or Antibody-Mediated Cellular Cytotoxicity Reaction

Type II reactions are cytotoxic reactions in which IgG or IgM bind to cell surface antigens, leading to complement activation or opsonization or activation of antibody-dependent cellular cytotoxicity and subsequent destruction of target cells [64]. This mechanism of allergy development is characteristic of drug-induced cytopenias (thrombocytopenia, anemia, leukopenia) due to drug binding to the surface of blood cells during transport but can also lead to the death of other cells.

It has been shown that the process of trogocytosis can lead to a decrease in cytotoxic reactions, resistance to biological therapy, and subsequent tumor evasion from immune surveillance [39,43]. Following administration of monoclonal antibodies (e.g., rituximab), cells expressing the FcγR, including monocytes, macrophages, neutrophils, and NK cells, can leach mAb-associated cell surface molecules from target cells through trogocytosis. This ultimately leads to a decrease in the effectiveness of mAb-based therapy. Consequently, during the process of trogocytosis, a decrease in the immune response to surface antigens, which underlies type II allergic reactions, is possible due to the removal of antigens from the cell surface and the development of anergy.

### 4.3. Type III or Immune Complex-Mediated Reactions

Type III is an immune complex hypersensitivity, as it develops due to the deposition of immune complexes in various organs and tissues [64]. Unlike type II, the allergen is soluble, and its complex with IgM and IgG is not fixed to target cells but is present in a fluid, often in the blood, and less commonly in tissue fluid. The deposition of immune complexes leads to the activation of immune system components, primarily the complement system, with the formation of anaphylotoxins C3a and C5a, which, in turn, are capable of activating immune cells. However, in the absence of certain complement components, especially C3, type III hypersensitivity also activates due to impaired clearance of immune complexes and their prolonged presence in tissues. Also, with the development of a type III reaction, a decrease in the levels of C3 and C4 in the blood is possible due to consumption [72]. III type of hypersensitivity is associated with serum sickness, serum-like syndrome, drug-induced vasculitis, and hypersensitivity pneumonitis.

Complement component C3 is known to bind to pMHC-II located on the surface of DCs, forming a covalent bond with the carbohydrate moiety of the MHCII α-chain [73]. Because C3 binding to the cell surface can cause cell damage, it is converted to inactive C3dg while remaining bound to pMHC-II. These pMHC-II–C3dg complexes are recognized by complement receptor 2 (CR2), which is highly expressed by marginal zone B cells. This results in trogocytosis, and marginal zone B cells become capable of presenting pMHC-II to T cells, which they do not generate themselves but receive from DCs. Thus, the absence or reduction in C3 levels in type III will result in decreased trogocytosis by marginal zone B cells.

### 4.4. Type IV or Cell-Mediated Reactions

Type IV is known to be the only type not associated with antibodies. The T-cell-mediated immune response develops 48–72 h after exposure to the antigen, but may not manifest until a couple of weeks later. The primary cells responsible for this response are T lymphocytes (CD4^+^ and CD8^+^), which trigger the release of cytokines, inflammation, and tissue damage [74].

#### 4.4.1. Type IVa—T1 Immune Response

Th1 helpers and the cells they activate (macrophages, CD8 T lymphocytes, NK cells, etc.) are involved in the development of this subtype [64]. This variant is responsible for the development of allergic contact dermatitis, celiac disease, erythema multiforme, and other diseases caused by infectious antigens, among other things. Since trogocytosis largely promotes polarization toward Th2 and reduces the formation of Th1 and secretion of IFNγ [47], the process of trogocytosis will likely lead to a weakening of this type of allergy. However, there is evidence of activation effector cells involved in type IVa in the process of trogocytosis [75]. It was shown that DCs that receive the pMHC-I can enhance the cytolytic activity of antigen-specific T cells. This may be necessary to increase survival and reduce mortality in mice bearing tumors. This effect is associated with the production of pMHC-I, which are expressed on the surface of tumor and virus-infected donor cells, and then transferred by DCs to recipients. The latter express trogocytosed molecules on their cell surface to activate the T-cell response. This mechanism provides an additional antitumor mechanism, and when this process is disrupted, the tumor escapes immune surveillance. However, it was shown that CD8^+^ T cells receiving pMHC-I from APCs or tumor cells can undergo fratricide [47]. A CD8^+^ T cell carrying pMHC-I presents the antigen to another CD8^+^ T cell, which triggers interferon gamma secretion, proliferation of other CD8^+^ T cells, and lysis of those CD8^+^ T cells that have pMHC. In type IVa, not only CD4^+^ and CD8^+^ αβT cells play a significant role, but also γδT cells, which are also capable of trogocytosis. It was shown that patients with an allergy to metals (in particular chromium) have a higher proliferation of CD8^+^CD25^−^, CD4^+^ and CD8 ^+^ cells, as well as γδT cells [76]. T cells are capable of presenting pMHCI complexes to allergen-specific CD8^+^ αβT cells through cross-presentation [77]. It has also been noted that CD1d^+^Vγ9Vδ2 T cells have the ability to produce Th-1 cytokines, particularly IFNγ [25], which may enhance the reactions underlying type IVa allergy.

Another example of the involvement of trogocytosis in type IVa is the spread of infection from uninfected cells to infected ones [78]. A macrophage can nibble off part of the membrane of an infected cell, including the MHCII along with the intracellular bacterium F. tularensis. The viability of both cells remained unchanged after transfer. Thus, F. tularensis bacteria can use this mechanism for dissemination, but, on the other hand, additional MHCII and other molecules received by cells can enhance the T cell response.

Thus, in type IV, trogocytosis can play a variety of roles, including positive (by enhancing the antitumor immune response and T cell response) and negative (by promoting pathogen dissemination).

#### 4.4.2. Type IVb—T2 Immune Response

Th2 cells, as well as ILC2, eosinophils, basophils, MCs, and other immune system cells, play a key role in the development of this subtype [64]. A key difference from type I is the long-term, persistent chronic inflammation caused by eosinophils.

This type contributes to a number of allergic diseases that develop according to type I, such as allergic rhinitis and atopic dermatitis, but can also develop via a non-IgE-dependent mechanism in eosinophilic esophagitis, eosinophilic bronchial asthma, and DRESS syndrome. Since trogocytosis can lead to cell activation and stimulation of the Th2 immune response, it can presumably enhance type IVb reactions; further research in this area is required.

#### 4.4.3. Type IVc—T3 Immune Response

Another subtype of the cellular response type is IVc, which develops through the T3 immune response, leading to the activation of neutrophils, with the participation of ILC3, Th17, and cytotoxic T cells type 17 (TC17) [64]. Diseases developing through this type include many autoimmune pathologies with neutrophilic inflammation. In the case of reactions to exogenous agents and the development of allergies, examples include acute generalized exanthematous pustulosis (AGEP) and atopic dermatitis. Studies have also found trogocytosis in the neutrophil population, with CD8 and additional TCR and CD3 transferred from T cells as a result of FcγR-mediated trogocytosis by neutrophils. It was shown that neutrophils can reduce immune responses and remove excess autoantibodies, which may be a protective mechanism [39]. However, in the case of trogocytosis by T helper cells, an increase in the T3 immune response may occur due to increased polarization towards Th17 [1].

### 4.5. Type V—Epithelial Barrier Defect

This type is associated with dysfunction and damage to barrier tissues, causing epithelial barrier cells and smooth muscle tissue to secrete alarmins, which in turn attract various immune system cells and promote inflammation [64]. It is caused by both a genetic predisposition (a mutation in the filaggrin gene) and the effects of allergens, pollutants, detergents, and other substances on barriers. Examples include atopic dermatitis, allergic bronchial asthma, allergic rhinitis, and other diseases that predominantly develop via the T2 immune response. Accordingly, as noted above, trogocytosis can enhance immune responses in this type. The possible role of trogocytosis in initiating this process following damage to barrier tissues has not yet been studied.

### 4.6. Type VI—Metabolic-Induced Immune Dysregulation

Type VI is metabolically induced immune dysregulation. An example is the development of bronchial asthma in obese patients [64]. Obesity is associated with negative effects on the body, leading to the development of type 2 diabetes mellitus, Alzheimer’s disease, vascular dementia, obstructive sleep apnea, atherosclerosis, heart failure, fatty liver disease, non-alcoholic steatohepatitis, osteoarthritis, as well as a shift in the immune balance towards the pro-inflammatory side [79,80]. It is known that obesity is accompanied by a chronic increase in the level of inflammatory cytokines, such as IL-6 and TNF. These pro-inflammatory cytokines can further stimulate macrophage infiltration into adipose tissue. Proinflammatory cytokines, osseous phase proteins, and factors produced by adipocytes themselves, such as leptin, lead to inflammation activation and disease development. Research has shown that macrophages can trogocytose aged adipocytes at the site of adipocyte-macrophage interaction via an “eat me” signal [81]. Trogocytized macrophages secrete IL-6 and MCP-1, which enhances adipose tissue inflammation. Thus, trogocytosis may amplify the reactions underlying type VI.

### 4.7. Type VII—Direct Cellular and Inflammatory Response to Chemical Substances

This type involves a direct cellular inflammatory response to chemicals [64]. It can develop with medications and certain foods. Examples include reactions to histamine liberators, nonsteroidal anti-inflammatory drugs, codeine, and other medications. The reaction to chemicals develops rapidly and without the prior sensitization required for type I. Due to the lack of need for immune cell interaction in this type, the role of trogocytosis as a key factor regulating the immune response is questionable.

Consequently, data on the role of trogocytosis in allergies are contradictory. Trogocytosis can develop with various types of hypersensitivity, primarily with those that follow the T2 type of immune reactions. However, given the fact that Treg cells trogocytize more actively than non-T-regulatory cells, the process of trogocytosis is likely to contribute more to the development of tolerance to the antigen.

## 5. Conclusions

Trogocytosis, as a form of cell interaction, is observed in both health and disease. Cells involved in the development of allergies are capable of trogocytosis. Through trogocytosis, cells can acquire new functions, participating in immune regulation. Thus, trogocytosis plays a crucial role in the immune system. The “nibbling” of various molecules by immune cells, tumor cells, and others, can lead to both beneficial and detrimental effects. In the case of immune cells, trogocytosis alters the direction of the immune response, either activating or suppressing cell activity. The possible role of trogocytosis in various immunopathological processes, in particular in allergies, has not been fully studied. On the one hand, trogocytosis promotes the polarization of the immune response towards T2, on the other hand, it suppresses the immune response through the generation of Tregs and their “biting off” molecules from the surface of APCs, which prevents the development of the immune response. However, given the fact that Treg cells trogocytize more actively than conventional T cells, the process of trogocytosis is likely to contribute more to the development of tolerance to the antigen. The exact mechanisms and role of trogocytosis in allergies remain unclear. Further research into trogocytosis may contribute to a better understanding of pathological processes and the discovery of new targets for therapy.

## Data Availability

No new data were created or analyzed in this study.
